# Risk factors for acute exacerbation of interstitial lung disease during chemotherapy for lung cancer: a systematic review and meta-analysis

**DOI:** 10.3389/fonc.2023.1250688

**Published:** 2023-10-11

**Authors:** Zhen Wang, Jiayu Bai, Yujia Liu, Peng Li, Guangyu Jiao

**Affiliations:** ^1^Department of Respiratory Medicine, Shengjing Hospital of China Medical University, Shenyang, China; ^2^Department of Rheumatology, The 1st Affiliated Hospital of China Medical University, Shenyang, China; ^3^College of Traditional Chinese Medicine, Liaoning University of Traditional Chinese Medicine, Shenyang, China

**Keywords:** lung cancer, interstitial lung disease, chemotherapy, acute exacerbation, risk factors

## Abstract

**Purpose:**

The aim of this study was to investigate the risk factors for acute exacerbation (AE) of interstitial lung disease caused by chemotherapy for lung cancer.

**Methods:**

We searched PubMed, Embase, and The Cochrane Library databases from the establishment of each database to April 2023. Eligible studies were included, and the data on risk factors related to AE caused by chemotherapy in interstitial lung disease were extracted.

**Results:**

A total of 878 articles were retrieved and 21 met the inclusion criteria. The studies included 1,275 patients with lung cancer combined with interstitial lung disease. The results of the meta-analysis showed four significant risk factors for AE of interstitial lung disease, namely age < 70 years (odds ratio [OR]: 1.98, 95% confidence interval [CI]: 1.05–3.72), forced vital capacity (FVC) (MD=-9.33, 95% CI: -13.7–4.97), usually interstitial pneumonia (UIP) pattern on computed tomography (CT) (OR: 2.11, 95% CI: 1.43–3.11), and serum surfactant protein D (SP-D) (SMD: 0.35, 95% CI: 0.03–0.67).

**Conclusion:**

When patients with lung cancer complicated with interstitial lung disease are aged < 70 years, have a UIP pattern on CT, have lower FVC values, and have higher serum levels of SP-D, chemotherapy should be carried out with care.

## Introduction

Interstitial lung disease (ILD) is a group of diffuse lung diseases that involve the lung interstitium and alveolar space, resulting in the loss of alveolar-capillary functional units. It is usually characterized by slow progressive respiratory insufficiency. However, some patients with ILD develop an acute exacerbation (AE), which is usually characterized by sudden progression and severe respiratory failure, and this condition is usually fatal for the patient.

Lung cancer is the most common primary lung malignant tumor and the leading cause of cancer death worldwide, and its mortality rate has been increasing. In recent years, although remarkable progress has been made in the diagnosis and treatment of lung cancer and ILD, the emergence of lung cancer combined with ILD has presented a difficult problem for clinicians. Patients with ILD are 7–14 times more likely to develop lung cancer than those without ILD. In fact, 4%–38% of patients with ILD also have lung cancer. Therefore, lung cancer is considered to be the most common complication of ILD (1). For patients who have advanced disease and are unable to undergo surgery, chemotherapy is the primary means of survival and symptom relief. However, most lung cancer patients with ILD are excluded from clinical trials because of the risk of AE of ILD caused by chemotherapy (2). Since the optimal treatment regimen for lung cancer complicated with ILD remains to be determined, it is essential to explore the risk factors for AE of ILD during chemotherapy for lung cancer.

Although existing studies have found some risk factors for AE of ILD during lung cancer chemotherapy, the conclusions are inconsistent, and the number of included patients is limited. Therefore, this systematic review and meta-analysis aimed to explore the risk factors for AE of ILD during lung cancer chemotherapy.

## Methods

### Literature search

The PubMed, EMbase, and The Cochrane Library databases were searched for studies on the risk factors for AE of ILD in patients with lung cancer complicated with ILD during chemotherapy. The search was performed from the establishment of each database to April 2023. Furthermore, the references cited in the included studies were traced to supplement the acquisition of relevant literature. Keywords in English included lung cancer, interstitial lung disease, chemotherapy, and risk factor.

### Inclusion and exclusion criteria

#### Inclusion criteria

Studies that met the following criteria were included: (1) All included patients were pathologically diagnosed with lung cancer and had imaging or pathological diagnosis of ILD; (2) All included patients received chemotherapy; (3) Foreign published studies on the risk factors for AE of ILD in patients with lung cancer patients receiving chemotherapy; (4) The language was English.

#### Exclusion criteria

We excluded following the studies: (1) Studies including patients receiving concurrent surgery or radiotherapy; (2) Repeated publication, case reports, conference literature, and review literature; (3) Inconclusive literature; (4) Documents with incomplete and inaccurate original data.

### Literature screening and data extraction

Two researchers independently completed literature screening and data extraction and cross-checked. If there was any disagreement, it was resolved by the two researchers and a third party after consultation. Data on the publication’s first author, publication year, region, study type, risk factors, and other relevant data were extracted.

### Literature quality evaluation

The Newcastle–Ottawa Scale (NOS) was used to evaluate the quality of case-control and cohort studies. The NOS evaluation content included four items of research object selection (4 points), one item of intergroup comparability (2 points), and three items of outcome measurement (3 points). The total score was 9 points, and studies with a score ≥6 were classified as high-quality research and rated as grade A. Studies with a total score < 6 were classified as low-quality research and rated as grade B. Methodological quality of the included studies were evaluated independently by two researchers. If there was any disagreement, it was resolved after consultation with a third researcher.

### Statistical analysis

RevMan5.3 software was used for data analysis. The Q test and I² statistics were used to evaluate the heterogeneity between the studies. When there was no significant heterogeneity, a fixed-effect model was used to combine the effect sizes; otherwise, a random effect model was used. Count data were represented using the odds ratio (OR) and a 95% confidence interval (CI), and the measurement data were represented using mean deviation (MD) and a 95% CI. Begg’s and Egger’s tests were used to evaluate publication bias.

## Results

### Characteristics of included literature

**Figure 1:**
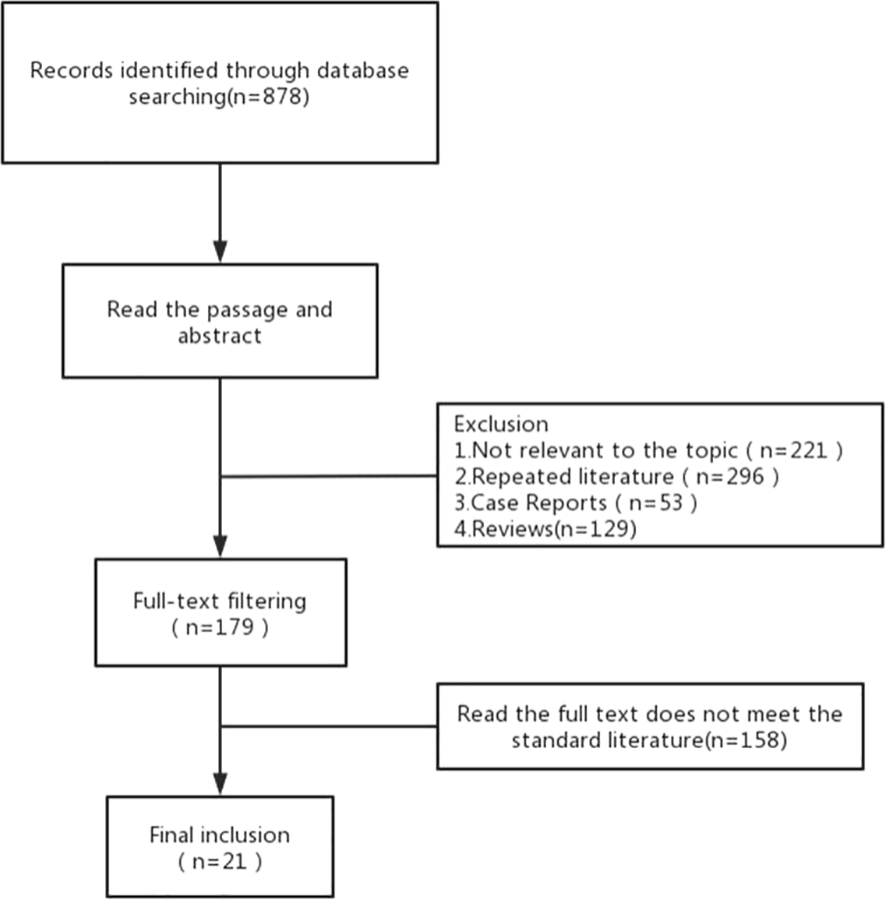
Flow chart of the identification of relevant studies.

A total of 878 articles were retrieved. After reading the title and abstract, 21 articles were included in the meta-analysis in strict accordance with the inclusion and exclusion criteria and were double cross-checked. The literature screening flow chart is shown in **Figure 1**. The 21 included studies were published between 2011 and 2022, and our study included a total of 1,275 ILD patients with lung cancer. ILD was mainly diagnosed by computed tomography (CT) and pathology. Finally, we focused on the risk factors for AE in ILD during lung cancer chemotherapy (**Table 1**).

**Table 1:** Characteristics of the included studies.

Author	Year	Country	Sample size	No. acute exacerbation n (%)	Diagnosis of interstitial lung disease	Risk factors															
						Sex	Age ≥ 70 years	Smoking history	Smoking quantity*	Clinical stage (IV and recurrence)	PS score (0–1)	UIP pattern	Use of immunosuppressive agents	IPF	CRP	SP-D	FVC, %	VC, %	KL-6	LDH	DLCo, %
Asai et al. (3)	2017	Japan	46	15	CT	√		√		√	√	√									
ITO et al. (4)	2021	Japan	81	27	CT	√				√	√	√	√								
KINOSHITA et al. (5)	2012	Japan	22	3	CT, Pathology	√		√		√	√							√	√	√	
Igawa et al. (6)	2022	Japan	24	7	CT	√		√			√	√									
Shirasawa et al. (7)	2019	Japan	31	7	CT	√		√			√				√	√			√	√	
Masuda et al. (8)	2018	Japan	35	9	CT	√			√	√	√	√							√		
Enomoto et al. (9)	2016	Japan	85	26	CT	√		√	√	√		√	√	√	√		√		√		√
SAIJO et al. (10)	2019	Japan	17	5	CT	√					√			√	√			√	√	√	
Akaike et al. (11)	2020	Japan	33	7	CT	√	√	√			√	√									
Hamada et al. (12)	2019	Japan	48	7	CT	√		√		√		√			√		√		√	√	
Kenmotsu et al. (13)	2011	Japan	109	24	CT	√	√			√	√	√									
Kanaji et al. (14)	2016	Japan	53	10	CT	√		√		√	√			√			√	√	√	√	
Watanabe et al. (15)	2015	Japan	35	5	CT	√	√				√	√									
Enomoto et al. (16)	2015	Japan	23	5	CT	√					√	√			√				√		
Kakiuchi et al. (17)	2017	Japan	64	4	CT										√	√		√	√	√	√
YOSHIDA et al. (18)	2013	Japan	52	6	CT	√	√				√										
Koda et al. (19)	2022	Japan	109	23	CT	√				√	√	√		√	√	√	√		√	√	√
NAKAO et al. (20)	2019	Japan	86	30	CT	√			√	√	√						√		√		
Otsuka et al. (21)	2022	Japan	35	8	CT	√		√			√	√			√			√	√	√	
Nishiyama et al. (22)	2020	Japan	105	12	CT	√	√				√	√				√			√		
Sekine et al. (23)	2022	Japan	182	30	CT	√		√	√	√	√	√	√	√							

smoking quantity (number of smoking packages * number of smoking years)

### Literature quality evaluation

A total of 21 articles were included, including 17 cohort studies and four case-control studies. The quality evaluation scores of cohort studies and case-control studies were 6–8, and the quality evaluation scores of the included literature were all ≥6. The quality evaluation score of seven articles was ≥8, indicating that the overall quality of the included studies was high (**Table 2**).

**Table 2:** Quality evaluation of included literature (Newcastle–Ottawa score).

Included literature	Research object selection	Comparability between groups	Result measurement	NOS score	Quality grade
Asai et al. (3)	3	1	2	6	A
ITO et al. (4)	4	2	2	8	A
KINOSHITA et al. (5)	4	1	3	8	A
Igawa et al. (6)	4	1	3	8	A
Shirasawa et al. (7)	4	1	2	7	A
Masuda et al. (8)	3	1	3	7	A
Enomoto et al. (9)	4	1	2	7	A
SAIJO et al. (10)	4	1	3	8	A
Akaike et al. (11)	4	1	3	8	A
Hamada et al. (12)	4	1	2	7	A
Kenmotsu et al. (13)	4	1	2	7	A
Kanaji et al. (14)	4	1	2	7	A
Watanabe et al. (15)	4	1	2	7	A
Enomoto et al. (16)	4	1	2	7	A
Kakiuchi et al. (17)	4	1	2	7	A
YOSHIDA et al. (18)	4	1	3	8	A
Koda et al. (19)	3	1	2	6	A
NAKAO et al. (20)	4	1	2	7	A
Otsuka et al. (21)	4	1	2	7	A
Nishiyama et al. (22)	4	2	2	8	A
Sekine et al. (23)	4	1	2	7	A

### Results of meta-analysis

#### Age

**Figure 2:**
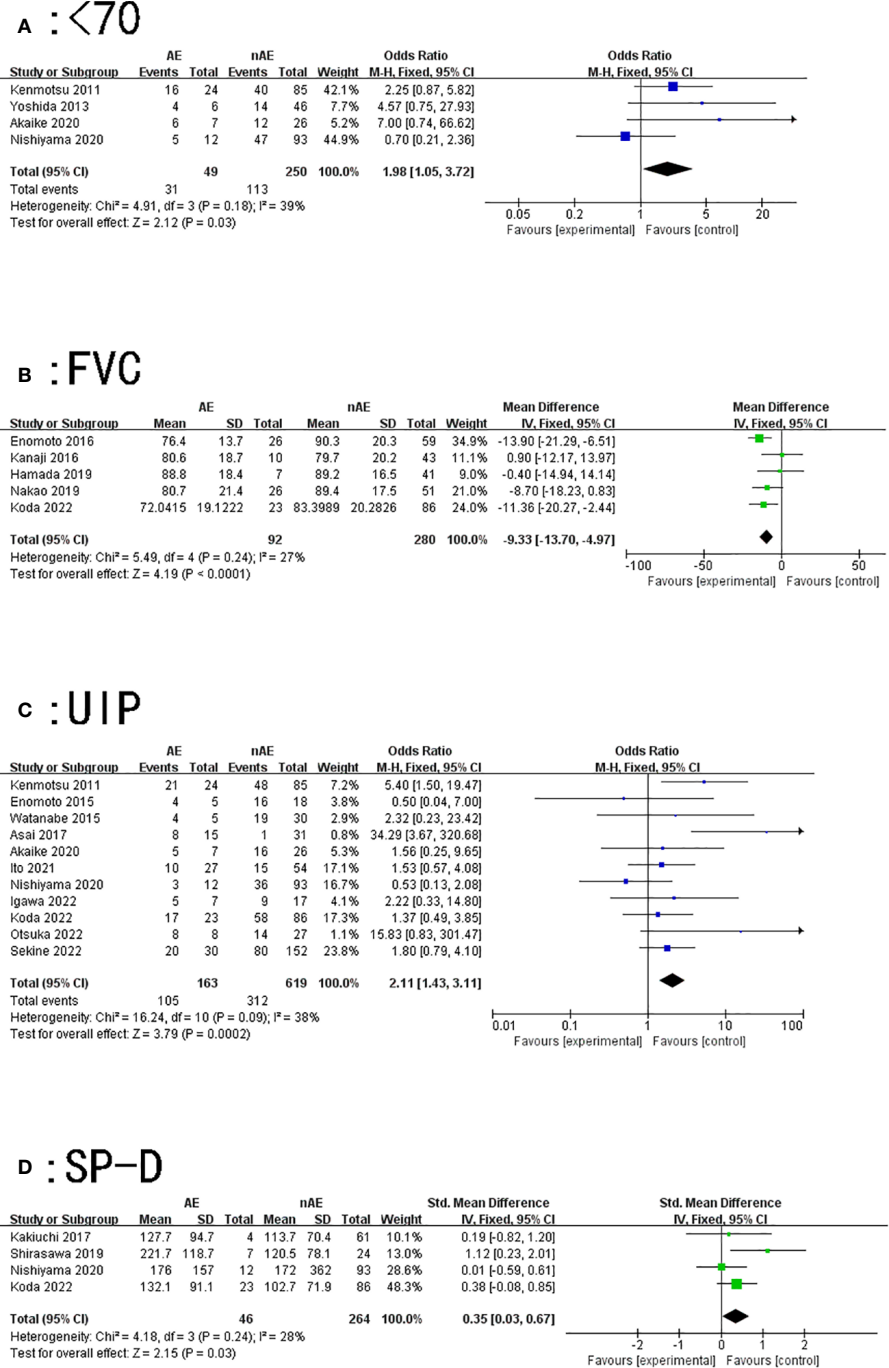
Forest plot of the potential risk factors for acute exacerbation of interstitial lung disease induced by lung cancer chemotherapy: **(A)** age < 70 years; **(B)** forced vital capacity (FVC); **(C)** usually interstitial pneumonia (UIP) pattern on computed tomography; and **(D)** surfactant protein-D (SP-D).

A total of four articles described the relationship between the occurrence of AE of ILD and age < 70 years during lung cancer chemotherapy. According to the results of the heterogeneity test (P=0.18, I^2 = ^39%), there was slight heterogeneity among the studies. The fixed effect model’s combined results (OR: 1.98, 95% CI: 1.05–3.72, P=0.03) demonstrated a significant difference (**Figure 2A**).

#### Pulmonary function test

Our findings suggest that patients with lung cancer with lower forced vital capacity (FVC) values were prone to AE of ILD while receiving chemotherapy (MD=-9.33, 95% CI: -13.7–4.97, P<0.0001) (**Figure 2B**).

Vital capacity (VC) (MD= -6.07, 95% CI: -14.66–2.53, P=0.17), and diffusing capacity for carbon monoxide (DLCo) (MD=2.08, 95% CI: -4.11–8.27, P=0.51) may not be risk factors for AE of ILD caused by chemotherapy. As there were only two articles on the detection of forced expiratory volume in 1 second among the included studies and no statistical significance was found, it is not discussed further in this article.

#### UIP pattern on CT

A total of 11 articles described the relationship between a UIP pattern on CT and AE of ILD induced by lung cancer chemotherapy. The results of the heterogeneity test (P=0.09, I^2 = ^38%) indicated that there was slight heterogeneity among the studies; the fixed effect model’s combined results (OR: 2.11, 95% CI: 1.43~3.11, P=0.0002) indicated a significant difference (**Figure 2C**).

#### Serum SP-D

A total of four articles described the relationship between SP-D and AE of ILD caused by lung cancer chemotherapy. The results of the heterogeneity test (P=0.24, I^2 = ^28%) showed that the heterogeneity between the studies was small; the fixed effect model’s combined results (SMD: 0.35, 95% CI: 0.03–0.67, P=0.03) demonstrated that the results were significant (**Figure 2D**).

This study finally included 16 risk factors for AE in lung cancer chemotherapy-induced ILD, of which four were significant, namely age < 70 years (OR=1.98, 95% CI: 1.05–3.72), FVC (MD: -9.33, 95% CI: -13.7–4.97), UIP pattern on CT (OR: 2.11, 95% CI: 1.43–3.11), and serum SP-D (SMD: 0.35, 95% CI: 0.03–0.67). There was no significant association for the remaining 14 potential risk factors, which were sex, smoking history, smoking quantity, clinical stage (IV and recurrence), PS score (0–1), use of immunosuppressive agents, idiopathic pulmonary fibrosis (IPF), c-reactive protein (CRP), VC%, Krebs von den Lungen -6 (KL-6), lactate dehydrogenase (LDH), and DLCo (**Table 3**).

**Table 3:** Meta-analysis of the risk factors for acute exacerbation of lung cancer complicated with interstitial lung disease during chemotherapy.

Risk factor	Number of articles	Sample size			Heterogeneity test			OR /MD/SMD	95%CI	P
		AE		Non AE	I^2^(%)	P				
Gender	13	177		697	4	0.41		1.53	0.83~2.83	0.18
Age<70	4	49		250	39	0.18		1.98	1.05~3.72	0.03
Smoking history	7	80		315	0	0.97		0.75	0.25~2.20	0.60
Smoking quantity	3	83		294	25	0.26		0.95	-4.67~6.58	0.74
Clinical stage (IV and recurrence)	8	142		484	19	0.28		0.97	0.65~1.46	0.89
PS (0-1)	12	169		670	0	0.53		1.12	0.69~1.83	0.64
Immunosuppressant use	3	83		265	0	0.69		0.65	0.27~1.53	0.32
UIP	11	163		619	38	0.09		2.11	1.43~3.11	<0.01
IPF	5	94		352	0	0.63		1.14	0.64~2.01	0.66
CRP	5	50		196	44	0.13		0.25	-0.18~0.69	0.26
SP-D	4	46		264	28	0.24		0.35	0.03~0.67	0.03
FVC%	5	92		280	27	0.24		-9.33	-13.7~-4.97	<0.01
VC%	3	21		89	36	0.21		-6.07	-14.66~2.53	0.17
KL-6	9	104		402	0	0.67		0.14	-0.08~0.36	0.21
LDH	7	63		258	47	0.08		0.11	-0.17~0.39	0.45
DLCo%	3	37		172	0	0.46		2.08	-4.11~8.27	0.51

#### Publication bias

**Figure 3:**
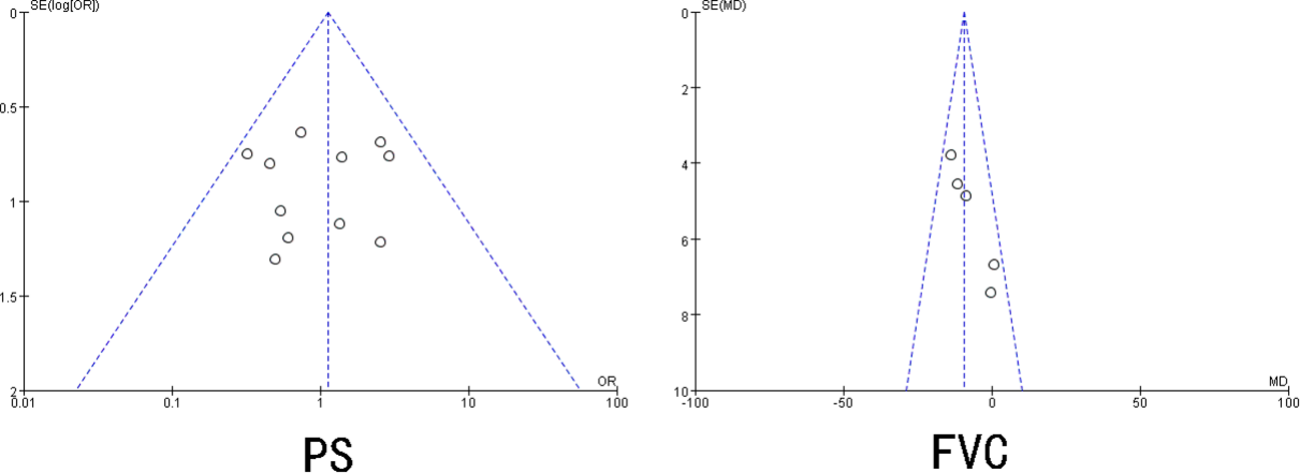
Positive funnel plot of publication bias.

Begg’s and Egger’s tests were adopted to check for publication bias in the included studies that reported ≥3 risk factors. The results showed that UIP pattern on CT, smoking, smoking quantity, sex, age < 70 years, use of immunosuppressive agents, IPF, clinical stage, KL-6, LDH, VC%, DLCo%, SP-D, and CRP had P>0.05, and the 95% CI was 0, indicating no significant publication bias in the included literature. The publication bias test for PS and FVC indicated the existence of a certain degree of publication bias (P < 0.05) (**Figure 3**).

## Discussion

In recent years, the incidence of lung cancer combined with ILD has gradually increased, and the prognosis is poor. For patients with advanced and inoperable lung cancer, chemotherapy is often the only treatment to prolong life; however, chemotherapy may be a fatal blow, inducing AE of ILD. Therefore, it is important for physicians to detect and confirm AE in a timely manner during chemotherapy. To the best of our knowledge, this is the first meta-analysis to investigate the risk factors for AE of ILD during chemotherapy for lung cancer. This study found four potential risk factors for AE in ILD during lung cancer chemotherapy: age < 70 years, lower FVC, UIP pattern on CT, and an increased level of serum SP-D.

Serological examination, chest CT, and determination of VC are important objective assessment methods in the treatment of patients with lung cancer. The pathogenesis of AE of ILD induced by chemotherapy remains unclear. Studies have suggested that most toxic effects are thought to be caused by direct cytotoxicity, and reactive oxygen species, growth factors, inflammatory cytokines, and angiogenic factors play an important role in inducing inflammation (24). The levels of these temporarily increase after chemotherapy, and the resultant inflammation is one of the causes of the AE. Therefore, increased levels of these cytokines may be associated with the activity of interstitial pneumonia (25, 26). SP-D is a known sensitive biomarker of interstitial pneumonia. The results of our meta-analysis showed, for the first time, that an increase in SP-D level significantly predicts AE of ILD during lung cancer chemotherapy. However, none of the included studies showed that SP-D had a role in predicting AE of ILD; this may be due to the small study population in the included studies. We found that patients with a UIP pattern on chest CT were more likely to have AE during chemotherapy. Asai et al. (3)believed that compared with alveoli with a UIP pattern, those with a non-UIP pattern had sufficient regulatory and clearance functions; therefore, lung cancer patients with ILD did not have AE during chemotherapy. It has also been shown that a UIP pattern has a more typical honeycomb lesion, which is associated with prognosis and fibrosis, and that patients with AE have significantly higher fibrosis than controls (27, 28). Therefore, patients’ chest CT images should be carefully evaluated before chemotherapy.

It is well known that a pulmonary function test is an effective means to assess the severity of lung lesions in patients. In a large study conducted by Song et al. (29), it was shown that a low FVC was a risk factor for AE of IPF, and other studies have shown that the decline of FVC within 6 months was one of the risk factors for AE of IPF (30). In this study, we found that a lower FVC was also important for predicting AE of ILD during lung cancer chemotherapy. Although the results of our meta-analysis showed that age (<70 years) was also a risk factor for AE of ILD during lung cancer chemotherapy, it was different from what we assumed. Older age was generally believed to be an important factor leading to disease exacerbation and even death. Kenmotsu et al. (13)suggested that these younger patients may be treated with multiple drugs for a longer period of time than older patients, and therefore have a higher probability of AE. On further analysis of the included studies, we found that they did not study the cycle and duration of chemotherapy by stratifying patients by age; therefore, further studies are needed to confirm this view.

In addition, some studies have found that PS score, CRP, LDH, albumin level, SUV_max_ of contralateral interstitial lesions on 18F-FDG PET/CT, sarcopenia, ground-glass attenuation (GGA) range and score, and positive autoantibodies are predictive (4, 7, 8, 11, 20, 22, 28, 29). However, these results were not confirmed in this study, and therefore need to be clarified by further research in the future. Among these predictive factors, GGA is pathologically related to inflammatory cell infiltration and interstitial fibrosis, and the immune response to chemotherapy drugs enhances inflammatory cell infiltration (30). Thus, the GGA observed on high-resolution CT may indicate an association with active ILD inflammation. Masuda et al. (8) demonstrated that the presence of GGA was a risk factor for chemotherapy-related AE of ILD. However, given the few studies available on this association, more clinical trials are needed in the future.

Pirfenidone and nintedanib are new anti-fibrotic drugs that can be used clinically as therapeutic agents for IPF; especially, nintedanib can prevent AE in IPF (31). However, there are no large-scale data to show whether these anti-fibrotic drugs can prevent AE of ILD during lung cancer chemotherapy. Otsubo et al. (32) conducted a randomized controlled clinical trial to determine whether nintedanib could prevent AE in patients with lung cancer complicated with IPF during chemotherapy. After 3 years of observation, although the primary endpoint of the study was not reached, it was still found that nintedanib combined with chemotherapy improved the overall survival rate of patients with lung cancer (33).

Although our study was conducted in strict accordance with the meta-analysis method, it has some shortcomings: (1) Almost all study populations were from Japan; therefore, the results may lack universality; (2) Most of the included studies were retrospective; there may be some bias, which did not reflect the actual situation of all patients; (3) The quality of the included literature was uneven, and the sample size of some studies was small, which may lead to the deviation of the results to some extent; (4) Some risk factors could not be effectively combined due to the few studies included, and the results of the meta-analysis were thus limited; (5) Different chemotherapy regimens have different risks of causing AE; however, this aspect was not examined in this study.

Despite these limitations, our meta-analysis provides new evidence for the risk of AE of ILD in lung cancer patients receiving chemotherapy. Age (<70 years), UIP pattern on CT, low FVC, and high SP-D level are more likely to cause AE of ILD in lung cancer patients during chemotherapy. Therefore, close attention needs to be paid to chemotherapy in clinical practice, and the treatment plan of such patients should involve a multidisciplinary cooperative approach. These risk factors can be considered in future to design high-quality studies with large samples and higher levels of evidence, and to provide references for more targeted intervention measures in clinical practice.

## Data availability statement

The original contributions presented in the study are included in the article/**Supplementary Material**. Further inquiries can be directed to the corresponding author.

## Author contributions

ZW and JB drafted the manuscript. ZW and GJ contributed to the study concept or design. YL and PL acquired the data. JB supervised and coordinated the study. All authors made a significant contribution to the work reported in terms of execution, analysis, and interpretation; took part in revising the manuscript; have agreed on the journal to which the article has been submitted and to be accountable for all aspects of the work. All authors contributed to the article and approved the submitted version.
