# Supplementary material for: Risk factors for acute exacerbation of interstitial lung disease during chemotherapy for lung cancer: a systematic review and meta-analysis
Source: Front Oncol. 2023 Oct 11;13:1250688. doi: 10.3389/fonc.2023.1250688 (PMC10598856; doi:10.3389/fonc.2023.1250688)
Supplement: Supplementary file 1 [file Table_1.docx]

Chemotherapy agents related to acute exacerbation of ILD

| **Chemotherapy**  **agents** | **No. of patients receiving**  **chemotherapy** | **No. of**  **AE-ILD (%)** |
| --- | --- | --- |
| Cisplatin | 154 | 9(5.8) |
| Carboplatin | 513 | 50(9.7) |
| Platinum agents | 787 | 75(9.5) |
| Paclitaxel | 230 | 24(10.4) |
| nab−paclitaxel | 106 | 14(13.2) |
| Docetaxel | 206 | 51(24.8) |
| Pemetrexed | 160 | 25(15.6) |
| Vinorelbine | 92 | 9(9.8) |
| Etoposide | 326 | 29(8.9) |
| Gemcitabine | 36 | 6(16.7) |
| S-1/UFT | 154 | 18(11.7) |
| Topotecan | 78 | 9(11.5) |
| Irinotecan | 99 | 3(3.0) |
| Amrubicin | 71 | 7(9.9) |
| Bevacizumab | 29 | 1(3.4) |
| Others^a^ | 14 | 2(14.3) |

a: Gefitinib/Erlotinib/Afatinib (n=7; AE=2); TS-1: tegafur-gimeraciloteracil potassium(n=7)
